# Oligometastatic disease – a renaissance for surgery?

**DOI:** 10.1515/iss-2023-0044

**Published:** 2024-08-02

**Authors:** Thorsten Goetze, Mickael Chevallay, Michel Dosch, Jordan Marcelis, Salah-Eddin Al-Batran, Stefan Paul Mönig

**Affiliations:** Institute for Clinical Cancer Research IKF, Frankfurt, Germany; UCT-University Cancer Center, Krankenhaus Nordwest, Frankfurt, Germany; Division of Digestive Surgery, University Hospitals of Geneva, Geneva, Switzerland; Oesophagogastric Surgery, Guy’s and St. Thomas Hospital, London, UK

**Keywords:** esophageal cancer, gastric cancer, cancer of the gastro-esophageal junction, oligometastatic disease, multimodal therapeutic approach

## Abstract

Half of the patients with esophageal cancer, cancer of the gastro-esophageal junction and gastric cancer present metastasis at the time of diagnosis. In addition, even patients originally thought to be free of metastasis will present metachronous metastasis in the course of the disease. These patients are considered incurable and current standard of care for metastatic esophageal, gastro-esophageal junction and gastric cancers is a systemic therapy without curative intention. However, patients presenting only a low metastatic load are now defined as oligometastatic disease and should benefit from an aggressive, multimodal therapy. We present here a review of recent publications investigating multimodal therapies for oligometastatic disease and showing that a systemic therapy combined with a resection of the primary tumor together with metastasis is associated with a better prognosis than a systemic therapy alone. We also give a precise focus on esophageal squamous cell carcinomas and adenocarcinomas of the gastro-esophageal junction and of the stomach. Interestingly, patients with oligometastatic cancer of the esophago-gastric junction can even be treated in curative intention with such a multimodal therapy as we present here in a short case report. In conclusion, new therapeutic strategies including multimodal approaches for oligometastatic disease have shown promising results in the last years and ongoing randomized prospective trials will provide us the evidence to include them in future European guidelines.

## Introduction

The presence of metastasis is often a barrier to surgical treatment in esophageal cancer and adenocarcinoma of the gastro-esophageal junction (GEJ). Indeed, up to 50 % of patients with esophageal and GEJ cancers have already metastatic disease at the time of diagnosis and are considered incurable with the standard of care in this situation being a systemic therapy [1], [2], [3]. In addition, initial non-metastatic disease can develop into metachronous metastatic disease during the course of their cancer history [4], [5], [6].

Among patients diagnosed with metastatic esophageal or GEJ cancers, oligometastatic disease is defined as an intermediate state between a loco-regional and a systemic disease [7]. Due to a low metastatic load with a circumscribed number of lesions, oligometastatic disease represents a potentially distinct and favorable tumor biology, which is a matter for further discussions and cannot be treated with a one fits all therapeutic concept [7].Currently, there is a major controversy regarding palliative and potentially curative treatment modalities in oligometastatic disease [8].

As part of research trials such as ongoing FLOT5-RENAISSANCE trial, treatment for oligometastatic disease should consist of a standardized systemic therapy combined with local therapies including surgical metastasectomy, ablation or stereotactic body radiation therapy (SBRT) and is possibly able to improve individual patient’s prognosis [9].

We present here a narrative review of the most recent literature on multi-modal therapies for oligometastatic diseases, with a particular focus on the role of surgery. The publications cited in this review have been selected by the authors for their relevance to the field, according to their own experience.

## Definition of oligometastatic disease

Currently, uniform criteria defining the maximum count of metastases and organs affected to be considered oligometastatic disease in esophageal and gastric cancers are lacking. Most of the studies define oligometastatic disease as a maximum of three lesions in one organ [10], [11], [12].

In 2020 the European Society for Radiotherapy and Oncology and European Organization for Research and Treatment of Cancer published their own classification for oligometastatic disease [13] and defined different scenarios. For example, an originally diffuse metastatic disease which is reduced to limited disease thanks to systemic therapy is further distinguished from a genuine and so-called upfront oligometastatic disease. Upfront oligometastatic disease is then further subclassified into a repeated oligometastatic disease (already existing history of oligometastatic disease) and *de novo* oligometastatic disease. *De novo* oligometastatic disease is further subdivided in synchronous and metachronous metastatic seeding. Furthermore, there is a differentiation into oligo-recurrence, -progression, and -persistence, to underline if oligometastatic disease occurred during a treatment-free interval or under ongoing systemic therapy [13].

In a study of Kroese et al. [7] 50 European expert centers were requested to discuss 15 real-life cases in their multi-disciplinary tumor boards in order to get data and find harmonization on the definition and treatment of oligometastatic esophagogastric cancer (OMEC project). In this trial, a consensus was reached on the definition of oligometastatic disease in esophagogastric carcinomas. The panel of experts defined oligometastatic disease as one or two metastases in either liver, lung, retroperitoneal lymph nodes, adrenal gland, soft tissue, or bone. In addition, the definition of oligometastatic remained appropriate at restaging after a median of 18 weeks of chemotherapy when no signs of tumor progression at the primary or the distant lesions were seen. Otherwise, if a progression was seen following systemic therapy these patients were no longer considered as oligometastatic.

The German AIO-FLOT study group [14] was able to establish criteria to identify patients with oligometastatic gastric or GEJ cancers who may potentially benefit from surgical resection after a systemic induction chemotherapy. These criteria are supported by data from the German prospective multicenter FLOT3 trial [14] and are the basis of the large multi-center randomized trial named Renaissance or FLOT5 trial [15]. Criteria defining oligometastatic disease in FLOT3 and 5 trials are also used in the German S3-Guidelines [16].

In FLOT3 trial [14], patients were stratified into three groups (A–C): A. clearly resectable gastric or GEJ cancers without evidence of systemic disease, B. so called oligometastatic cancers and C. diffuse metastatic patients. Oligometastatic patients in FLOT3 trial were defined as follow: Metastatic lymph node involvement at distant intra-abdominal lymph nodes only or/and a maximum of one metastatic organ system affected, defined as follows: up to five liver metastases, no signs of macroscopically detectable carcinomatosis (peritoneum or pleura), and good performance status (ECOG≤1). Metastatic patients not in the frame of the mentioned criteria were considered as so called diffuse metastatic patients.

The definition of oligometastatic disease in FLOT5 trial is derived from FLOT3 trial with some modifications and precisions. Oligometastatic disease has to be considered in the case of: 1. Metastatic infestation of retroperitoneal lymph nodes only (e.g., para-aortal, intra-aorto-caval, parapancreatic or mesenterial lymph nodes) (Note: duodenum invading gastric cancer, infestation of retropancreatic lymphatic nodes is not regarded as M1-disease) and/or 2. Infestation of a maximum of one organ combined with or without positive retroperitoneal lymph nodes according to the following criteria:

- Localized potentially resectable peritoneal seeding according to stage P1 of the “Japanese Research Society for Gastric Cancer” – classification, also allowed within the trial, a so called Peritoneal Cancer Index (PCI) according to Sugarbaker-definition of ≤6 (of note, patients with a Japanese P-score 2 or 3, but PCI≤6 are still eligible for trial inclusion) or
- Liver: not more than five metastatic lesions with the potency to be surgical R0-resectable or
- Lung: potentially surgically curable unilateral involvement or
- Uni- or bi-lateral ovarian Krukenberg metastatic lesions in the absence of a further macroscopic peritoneal seeding or
- Uni- or bi-lateral metastases of the adrenal gland or
- Extra-abdominal metastatic lymph node seeding e.g., supraclavicular, or cervical lymph node infestation or
- Localized bone infestation (respecting one radiation field) or
- Other metastatic infestation considered oligometastatic by the investigator and central review committee.

## Oligometastatic disease in esophageal squamous cell carcinoma

Especially in unresectable recurrent or metastatic esophageal squamous cell carcinoma (ESCC) most guidelines recommend chemotherapy [17], 18]. Despite the progress made in implementing new drugs, the duration of response to modern therapies is relatively short. Furthermore, it is acknowledged that a subset of patients with limited metastatic disease and a lower tumor burden can potentially benefit from radical and extensive local treatment strategies, which may result in longer-lasting response rates [19].

To date, in entities like colorectal and lung cancers, surgical resection alone or combined with interventional ablation of limited hepatic metastases is an accepted practice [20]. Concerning ESCC, there is still a large debate on how to manage oligometastatic patients. Several trials with only small patients numbers have shown that resection of metastases for esophageal cancer in a very selected patients subset with low tumor burden have shown promising results [21], [22], [23], [24]. Currently there is no consensus on the best possible treatment for patients with low tumor burden and recurrent ESCC. Therefore palliative chemotherapy including immunotherapy is the only proven standard of care in formally palliative patients treated with according to the Checkmate 648 protocol [19], 25], 26]. The discussion needs to be taken up again in the light of the recent results of the Keynote 590 and the Checkmate 648 studies investigating chemo-immunotherapy including an immunotherapy combined to a doublet chemotherapy with for example pembrolizumab or nivolumab [27], 28]. Checkmate 648 showed that both first-line treatment with nivolumab plus chemotherapy and first-line treatment with nivolumab plus ipilimumab resulted in significantly longer overall survival than chemotherapy alone in patients with advanced ESCC. In conclusion, immunotherapy can improve prognosis of patients with metastasis and should be integrated in oligometastatic concepts.

Metastatic patients represent a heterogeneous population depending on the status of the primary tumor, the disease-free interval, the number and the size of the lesions, and the performance status. These patients differ a lot from each other, however they are treated with a one fits all concepts including a systematic therapy due to the lack of large, randomized trials in ESCC with oligometastatic disease.

Although the prognosis for diffuse metastatic disease is poor, patients with oligometastatic disease may still have a chance of cure thanks to less aggressive tumor biology. These patients should be treated with new therapeutic concepts including a local therapy combined to systemic therapies using new drugs like immunotherapy. Ichida et al. reported that metastatic liver resection for patients with fewer than three lesions resulted in a 5-year overall survival rate of 30–50 % [21]. A further study was able to show significantly better outcome for patients with resection of the single metastases than for those who did not receive additional surgical intervention (3-year OS, 64.3 % vs. 10 %) [29]. In general, Ghaly et al. showed that there is no significant difference for the various local techniques used for single recurrent lesions of esophageal cancer in regard of overall survival [30].

The study of Liu et al. showed that the prognosis of patients with oligometastatic disease in ESCC was different from patients with diffuse metastatic disease. In this study, SBRT combined to chemotherapy or not was used for well-selected oligometastatic patients and achieved similar results to radical therapy for locally advanced ESCC. However, there were several limitations in the mentioned trial: it was a single-arm trial with a small sample size and selection bias, as well heterogeneity in population. Heterogeneity is often influenced by the fact that there is no real consensus about the definition of what is oligometastatic esophago-gastric cancer, especially in ESCC-disease.

## Oligometastatic disease in adenocarcinomas of the gastro-esophageal junction and of the stomach

Treatment of adenocarcinomas of the gastro-esophageal junction (GEJ) and gastric cancer is largely concomitant regarding the treatment strategies. Surgery for metastatic GEJ and gastric cancers in curative intention remains an experimental treatment with the need for individual patient’s decision. Surgery in curative intention has been evaluated in retrospective cohort studies for patients with oligometastatic disease [31]. Results indicate that surgery could provide a possible benefit for selected patient’s subpopulation, including patients aged 70 years or less with only one metastatic site (lymph nodes or liver) [32] and excellent response to an induction preoperative chemotherapy [33], 34], or patients with liver metastases and a complete liver metastasectomy [35]. However, hard criteria or data supporting these individual strategies are lacking.

Data from a German research team identified D3-lymph nodes as an independent predictor for survival based on data of 48 gastric cancers with primary gastric resection [36]. Japanese investigators showed data from a cohort of 16 gastric cancers with positive para-aortal lymphatic node involvement who received a curative intended surgical resection after induction with a triplet systemic therapy consisting of a taxane, platinum and fluoropyrimidine. The 2-years overall and the relapse-free survival in the study of our Japanese colleagues were quite promising with an overall survival (OS) of 93.8 % and recurrence free survival (RFS) of 75.0 % [37]. These data were supported by individual case reports [38], 39] in well selected patients subpopulations underlining a possible benefit of multimodal concepts in oligometastatic disease of upper-GI adenocarcinomas.

## New therapeutic strategies for oligometastatic disease

Oligometastatic disease should be treated using a multimodal therapeutic strategy combining chemotherapy, immunotherapy, and targeted therapy either to maintain a resectable state for oligometastatic disease or to diminish the metastasis size to obtain a complete resection configuration [40]. We cite here several recent publications that have led to breakthroughs in the treatment of oligometastatic disease in gastric and GEJ cancers.

Non-curatively treated patients included in the so called Dutch Gastric Cancer Trial [32] were evaluated for potential benefits of palliative surgery. A total of 26 % of the patients were found to present an incurable tumor at the time of laparotomy. Dutch colleagues proceeded then either to explorative laparotomy only, gastro-enterostomy or to the palliative tumor resection. Interestingly, survival was shown to be better in patients with tumor resection (8.1 vs. 5.4 months; p<0.001). Notably, patients with only one metastatic site have shown a significant survival benefit after resection (10.5 vs. 6.7 months; p=0.034) compared to patients with more than one metastatic site (5.7 vs. 4.6 months; p=0.084). The most significant improvement thanks to resection was seen in patients younger than 70 years combined to only one metastatic site, indicating that other factors besides tumor burden influence outcome in oligometastatic disease and that patients with low tumor burden must be fit enough for a multimodal concept including resection of primary tumor and definitive therapy of all lesions.

Kroese et al. showed in a multi-center trial about oligometastatic esophagogastric cancers [41], that a multimodal therapeutic concept combining local and systemic therapy leads to improved overall survival when compared with systemic therapy alone. In this trial, patients with different cancer types were included, however most of the patients had esophageal cancer (73 %) with adenocarcinoma histology (79 %) and metachronous oligometastatic disease 52 %).

The Spanish AGAMENON national observational registry [42] published data for surgical resection of metastatic disease for esophagogastric cancer. AGAMENON analysed data from 32 centres in 1,792 subjects. 5 % (n=92) of the 1,792 esophagogastric cancer patients received resection of metastases. Also AGAMENON showed improved survival after selection of patients for surgery, HR 0.34 (95 % CI, 0.06–0.80, p=0.021) but median OS after surgery was only in the range of current pure systemic therapy trials with an overall survival 16.7 months (95 % CI, 12.5–22.4) e.g. TOGA- or Checkmate 649-trial [43], 44] The 1- and 3-year relapse rates following R0 resection were 58 % and 65 %, respectively, the median time to recurrence since resection of all metastases was up to 8.4 months (95 % CI, 7.6–23.7) The Spanish colleagues summarized that esophagogastric cancer patients with only a limited number of metastases can benefit from surgical strategies. Nevertheless, absolute survival data were not better for patients with systemic in current landmark studies, with additional risk of surgery.

The ongoing discussion highlights the necessity for randomized trial data in the context of oligometastatic esophagogastric cancers.

A systematic review and pooled analysis by Markar et al. [45] investigated the influence of surgical resection of hepatic metastases regarding long-term survival, morbidity, and mortality from gastric cancer patients. They performed a systematic literature search between 1990 and 2015 and included only publications with a minimum of 10 patients with liver surgery for metastatic gastric cancer with hepatic disease only, focusing on overall survival as primary outcome. Other factors like e.g., multiple vs. single hepatic lesions or metachronous vs. synchronous disease were also investigated regarding their influence on survival. The analysis included 39 studies with a median of 21 (range 10–64) liver resections. Liver surgery was associated with a median 30-day morbidity of 24 % (0–47 %) and mortality of 0 % (0–30 %). The median survival after 1-, 3- and 5-years was 68 %, 31 %, and 27 % respectively and even better in Far Eastern compared with trials form Western world; 1-year (73 % vs. 59 %), 3-year (34 % vs. 24.5 %), and 5-year (27.3 % vs. 16.5 %). Overall, hepatic resections improved overall survival significantly (HR=0.50; p<0.001) especially in cases of single liver lesions compared with multiple hepatic metastases (odds ratio=0.31; p=0.011), implementing once more that after a proper selection of patients there might be a role for surgery in well-defined subgroup of patients with low tumor burden. Especially liver limited disease maybe another special subgroup.

Published in 2016, the Asian phase III REGATTA study investigated the superiority of gastrectomy followed by chemotherapy vs. chemotherapy alone with respect to overall survival in patient with incurable advanced gastric cancer [46]. Unfortunately, the REGATTA study could not show any survival benefit of gastrectomy followed by chemotherapy compared to chemotherapy alone. These results might be related to the fact that metastases were not resected in contrast to the FLOT3 and FLOT5 studies for example.

**Figure 1:**
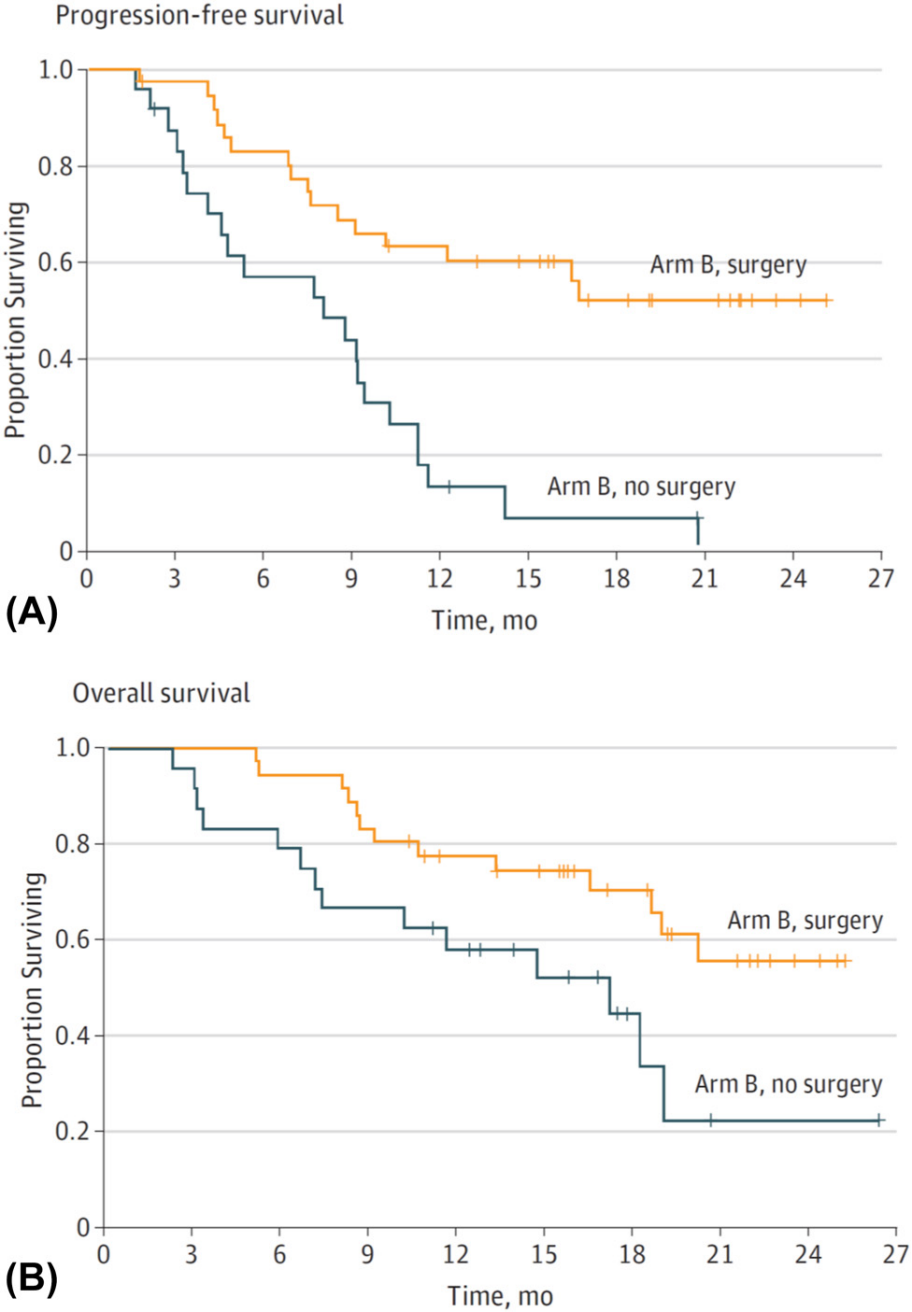
Kaplan–Meier analysis of progression-free survival (A) and overall survival (B) in patients with limited metastatic disease (arm B) who underwent surgery and no surgery. Crosshatching indicates censored data. Images are from ref. [14] with the kind permission of the authors.

In the FLOT3 trial mentioned above [14], patients without signs of systemic disease (arm A) received induction with four cycles of FLOT-therapy followed by a radical curatively intended tumor surgery followed by four cycles of adjuvant FLOT-regimen, in accordance with the FLOT4 landmark trial that followed based on this concept and established FLOT as the standard of care in the perioperative therapy of esophagogastric adenocarcinoma [47], 48]. Patients with oligometastatic disease (arm B) also received four cycles of FLOT followed by tumor surgery, including resection of metastases, if technically feasible. If surgery was feasible, a so-called adjuvant or additive FLOT-therapy was performed. Patients with diffuse metastatic cancers (arm C) received eight cycles of FLOT-regimen without the option of a curative or curatively intended surgery. In this group of patients, surgical techniques were limited to e.g. palliative bypass surgery. 25 % of patients in FLOT3 trial showed oligometastatic disease, out of them half underwent surgery after induction with four cycles of FLOT chemotherapy. Oligometastatic patients with radical surgery showed survival benefit with a median overall survival of 31 months compared with only 16 months in oligometastatic patients without radical surgery (Figure 1) [14]. These data provided the rational for the Renaissance/FLOT5 trial [15], 49].

**Figure 2:**
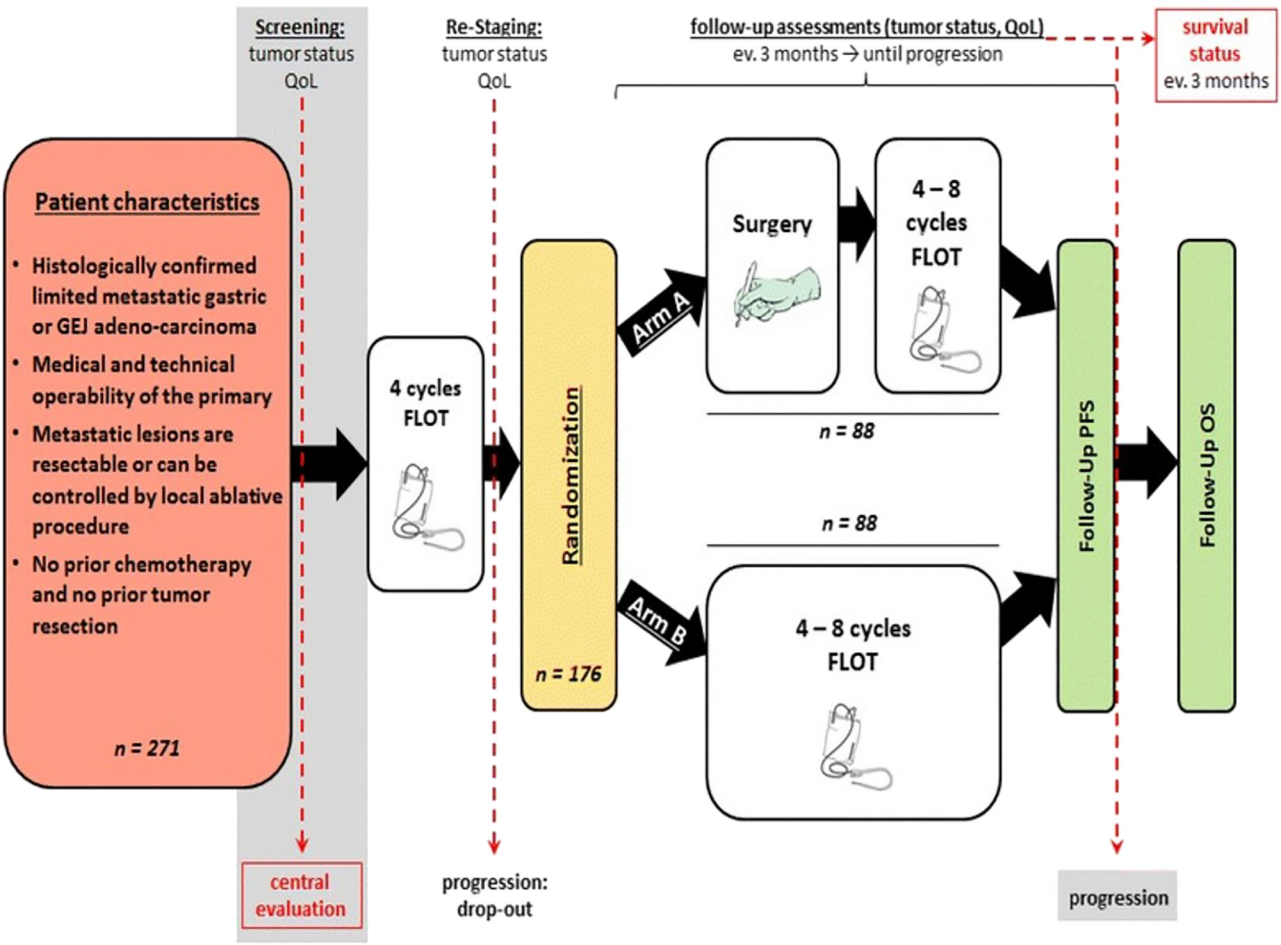
FLOT5 trial started in 2016 and includes patients with oligometastatic gastric or GEF adenocarcinomas. After four cycles of FLOT, patients are randomized in surgery arm or chemotherapy alone arm. Patients with surgery were admitted to four cycles of adjuvant FLOT. Image is cited from Al-Batran et al. [14], BMC Cancer with the kind permission of the authors.

FLOT5 trial is a prospective, multi-centre, randomized, investigator-initiated phase III trial study (Figure 2). FLOT5 trial is currently investigating the effect of a standardized induction chemotherapy with FLOT schema (+/− trastuzumab or nivolumab, depending on the HER2 and PD-L1 status) in therapy naïve oligometastatic patients in combination with curatively intended gastrectomy/esophagectomy plus resection of all metastatic sites or local ablation, radiation procedures with the goal to achieve a R0-situation at the primary as well all as metastatic sites (Figure 2).

Only patients with an upfront oligometastatic disease can be included. Patients with primary diffuse seeding showing a good response to chemotherapy, so called induced oligometastatic, and patients with tumor recurrence are not candidate to FLOT5 trial.

Due to the randomized nature of the study after four cycles of FLOT and restaging of patients in cases without signs of progressive disease, patients are randomized in a 1:1 fashion into radical surgery plus adjuvant systemic FLOT continuation or in a non-surgical arm, the standard arm with continuation of FLOT regimen only without radical surgery.

FLOT5 will be able to provide us with sufficient data in the adenocarcinoma subgroup of OMD esophagogastric cancer patients.

## Case report

**Figure 3:**
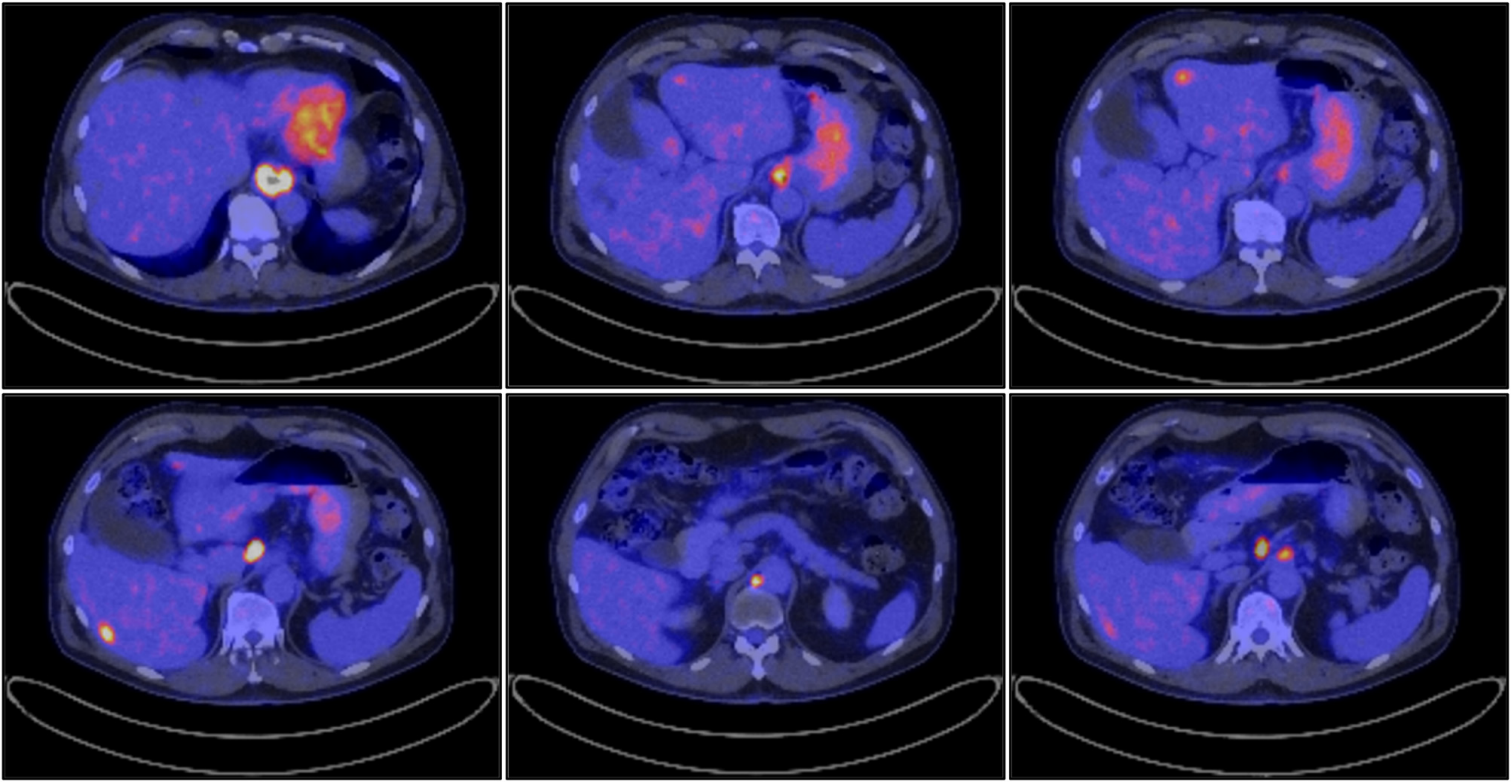
PET-CT showing mediastinal lymph node metastasis, hepatogastric lymph node metastasis and liver metastasis in segment II (8 mm), III (13 mm) and IV (12 mm and 5 mm) at the diagnosis. Images are cited with patient’s consent.

A 57-year-old male patient in good health without comorbidities was diagnosed with an adenocarcinoma of the gastro-oesophageal junction (GEJ) Siewert I with loco-regional and distant (mediastinal) lymph node metastases and four liver metastases in segments II (8 mm), III (13 mm) and IV (12 mm and 5 mm), initially classified stage IV (T3N2M1) (Figure 3).

Therapeutical strategy was discussed at our multidisciplinary tumor-board and the patient benefited first from two cycles of FLOT (fluorouracil plus leucovorin, oxaliplatin and docetaxel) regimen followed by restaging using PET-CT scan and MRI of the liver.

The PET–CT scan showed significant response in both mediastinal and hepato-gastric ligament lymph node metastases and a decrease in the primary tumor was observed. The MRI of the liver presented congruent results with the PET-CT scan showing a size reduction of liver metastases.

The patient underwent six additional cycles of FLOT chemotherapy according to the RENAISSANCE trial including Herceptin due to the presence of a HER2^+^ status from April to June 2021.

After a total of eight cycles of FLOT chemotherapy, a second restaging was performed showing the disappearance of the liver and lymph node metastases enabling a curative treatment.

A Ivor-Lewis esophagectomy including a two-fields lymphadenectomy and gastric tube reconstruction with intrathoracic anastomosis was performed. At the same time, an intraoperative ultrasound of the liver was done and showed no residual metastasis. Resection of liver segment III at the site of the former biggest metastasis was performed to ensure clearance of the largest metastatic lesion. Final pathologic findings of the resected specimen, including primary tumour and liver resection, showed no evidence for residual cancer but signs of complete pathological response, therefore staged as ypT0 N0 (0/44) L0 V0 Pn0 R0.

At 24 months after definitive treatment, the patient was doing well, and follow-up imaging showed no sign of recurrence. This example suggests that a multimodal therapy could be a promising strategy for the future treatment of oligometastatic disease of adenocarcinoma of the GEJ in curative intention.

## Conclusions

In conclusion, treatment for patients with esophageal, GEJ and gastric cancers presenting with limited “oligo-” metastatic disease remains controversial and a matter of ongoing research trials. Currently, no treatment standards are established and the recent ESMO guidelines and German S3 Guidelines do not recommend surgery for metastatic disease [16], 18]. However, treatment of oligometastatic disease should probably be more aggressive and randomized prospective trials like the German RENAISSANCE/FLOT5 trial will provide us more evidence in the next years about the indication of a multimodal therapy including surgical resection of primary tumor and metastasis.
